# Paper Ageing: The Effect of Paper Chemical Composition on Hydrolysis and Oxidation

**DOI:** 10.3390/polym13071029

**Published:** 2021-03-26

**Authors:** Edyta Małachowska, Dominika Pawcenis, Jacek Dańczak, Joanna Paczkowska, Kamila Przybysz

**Affiliations:** 1 Institute of Wood Sciences and Furniture, Warsaw University of Life Sciences—SGGW, 159 Nowoursynowska Str., 02-787 Warsaw, Poland; 2 Natural Fibers Advanced Technologies, 42A Blekitna Str., 93-322 Lodz, Poland; Jac.danczak@gmail.com (J.D.); kamila.przybysz@interia.pl (K.P.); 3 Faculty of Chemistry, Jagiellonian University, 2 Gronostajowa Str., 30-387 Kraków, Poland; pawcenis@chemia.uj.edu.pl (D.P.); lojewska@chemia.uj.edu.pl (J.P.)

**Keywords:** cellulose degradation, cellulose depolymerisation, hydrolysis, oxidation, crystallinity, paper ageing

## Abstract

The degradation of cellulose is an important factor influencing its mechanical, optical, physical, and chemical properties and, hence, the lifetime of paper in libraries and archival collections. Regardless of the complexity of the paper material, the main chemical pathways for its degradation are hydrolysis and oxidation. This study presents an overview of the analytical techniques employed in the evaluation of the hydrolysis and oxidation processes; these techniques include size-exclusion chromatography, Fourier-transform infrared and ultraviolet–visible spectroscopy, and X-ray diffraction. This paper aims to determine the extent to which these instrumental methods are useful for studying the aforementioned processes and for which lignin contents. It also highlights how atmospheric humidity could affect the cellulose structure in paper containing lignin. It was found that humidity causes significant changes in the cellulose chain lengths and that a high lignin content in paper could suppress some cellulose degradation pathways. This knowledge can be applied to developing strategies and selective chemical treatments preventing the consequences of paper ageing.

## 1. Introduction

The industrial revolution that took place around the 19th century, involving the papermaking industry among other sectors, advanced the popularisation of prints. Simultaneously, both the introduction of the semi-finished product obtained from wood (groundwood pulp) and the addition of alum in a new technological process achieved dramatic results, such as chemical destabilisation of the cellulose polymer [1,2]. It is claimed that destruction of the cellulose polymeric structure, as well as lignin, is generally caused by acidic hydrolysis of the glucopyranose rings and oxidation [3,4,5].

The hydrolysis of a cellulose macromolecule involves the breaking of the β(1→4) bonds between particular D-glucose units [6]. Hydrolysis results in cellulose degradation, during which polysaccharides are degraded to form oligosaccharides and monosaccharides. The shortening of the cellulose chain is expressed in terms of reduction in its degree of polymerisation (DP) [7]. Importantly, the glycosidic bonds in cellulose could only ensure its stability in a slightly alkaline or neutral environment while the increase in the hydronium concentration in an acidic medium could accelerate the hydrolytic process of glycoside bond breakages [8]. Apart from the initial pH level of the paper, the rate of hydrolysis could be determined by the temperature and presence of water vapour in the paper [9,10,11]. To initiate hydrolysis in paper with even a slight increase in temperature, a minimum amount of water (5%) in the paper, under normal conditions, is sufficient (in the case of paper, the temperature is 23 ± 1 °C, and the relative humidity is 50%). The mechanism of hydrolysis is relatively well known and described in the available literature [12,13].

The oxidation process, depending on the temperature, plays an important role in the degradation of paper [14,15]. At room temperature, the rate of paper degradation due to oxidation is low compared to the destruction caused by the acid hydrolysis process [16]. However, oxidation is a much more complex process than hydrolysis, although it is rarely described in the literature [1,17].

Further, the stable products of cellulose oxidation reaction are the carbonyl groups. The products of oxidation may also include primary alcohol groups and two secondary alcohol groups in each glucose unit as well as an aldehyde group, which is a cellulose functional group, at the end of the chain. Moreover, oxidation may also occur selectively. The primary alcohol groups on the C(6) atom may be oxidised into an aldehyde group and further into a carboxylic group. Further, the secondary alcohol groups on the C(2) and C(3) atoms may be oxidised into ketone groups. Consequent to the breakage of the carbon atom bonds, the destruction of the glucopyranose ring could occur with the formation of aldehyde groups which may, in turn, be oxidised to carboxyl groups. The oxidation of the hydroxyl group on the C(1) atom of the final glucose unit and the breakage of the glucopyranose ring in the adjacent position are possible [18,19]. The oxidation of the functional groups in glucose residues generally occurs simultaneously with breakage of the glycosidic bonds, which contributes to the lowering of the DP as well as to the durability of the cellulose materials in these residues. The advancements in the oxidation process and consecutive reactions may also result in the crosslinking of the neighbouring macromolecules through hemiacetal and ester bonds [20].

Compared to cellulose, lignin is much more sensitive to oxidation by atmospheric oxygen. Thus, paper containing large amounts of lignin, hemicelluloses, and additives is generally very susceptible to oxidation as well as hydrolysis and potential degradation processes, as confirmed by numerous studies [21,22]. Consequently, at the initial degradation stage, lignin may function as a shield for cellulose. In a wider perspective, radicals and active forms of oxygen as well as carboxyl groups, being the products of the lignin oxidation process, will reduce the pH of paper and intensify the whole cellulose degradation process.

Hydrolysis and oxidation processes are dependent on each other and catalyse each other [23]. The formation of carbonyl groups because of oxidation weakens the closest glycosidic bonds, thus making them more susceptible to hydrolysis [24,25]. Hydrolysis of the glycosidic bonds results in the creation of new end-groups (reducing) which are susceptible to oxidation. Moreover, water, being the byproduct of cellulose oxidation, is a substrate for hydrolysis which enables the transportation of protons, radicals, and oxygen active forms within the paper structure [5]. Oxidation, therefore, contributes to the creation of carboxyl groups, thus promoting acid hydrolysis [18].

The progress of hydrolysis and oxidation could result in changes in each level of the fibre structure. One of these changes is recrystallisation, which is demonstrated by the reorganisation of the macromolecules of cellulose into a more ordered structure. Although recrystallisation is not a chemical process, it can affect hydrolysis and oxidation processes. Considering this, recrystallisation should be included in assessing the degradation states of papers.

Degradation caused by hydrolysis and oxidation processes concerns a vast majority of libraries and archive collections. To improve the existing methods of conservation and rescue the collections of archival or historical values which have not been destroyed by acid-catalysed degradation, detailed studies on the mechanisms of the ageing processes are required [26]. Nondestructive or microdestructive analytical methods for the effective assessment of paper-ageing processes and their expected durability are especially significant. The most common and widely employed methods, consisting of the measurement of the colour, acidity [27,28], and tensile properties [29,30] of paper, do not fulfil the above requirements. Comprehensive estimation of the degradation of paper requires several complementary methods, which would be able to grasp the different aspects of paper destruction, from the mechanical to chemical aspects. Here, we focused on the chemical effects of deterioration. Viscometry, a classic analytical method which is inexpensive and relatively easy to perform, was employed for the estimation of cellulose depolymerisation. However, viscometry fails in the analysis of paper containing lignin. Therefore, other techniques, like size-exclusion chromatography (SEC), are recommended [27]. Consequently, SEC was employed in this work to study the changes in the DP of papers characterised by different lignin contents. Further, the process of oxidation was monitored using molecular spectroscopy techniques, such as Fourier-transform infrared (FT–IR) and ultraviolet–visible (UV–Vis) range spectroscopies. The X-ray diffraction (XRD) technique was employed to evaluate the degree of cellulose crystallinity in tested papers. The selection of methods allowed for the assessment of paper degradation states by the characterisation of the depolymerisation process, development of carbonyl groups, and recrystallisation of cellulose. Here, we attempted to examine the following issues:

- the extent to which increased humidity could affect the changes in the cellulose structure;
- the Kappa numbers for which the instrumental methods are applicable.

To achieve these objectives, artificially aged papers with differential lignin contents were investigated. The papers were produced at a neutral pH to eliminate the impact of acidity on the rate of degradation processes. Previous research and experience have shown that the rate of acid hydrolysis depends on the initial pH value [8]. The accelerated ageing conditions were adjusted to achieve the maximum effect (climatic chamber RH 59%, 90 °C) and also to compare it with results for conditions obtained in a dry atmosphere (RH 0%, 90 °C). Accordingly, with all the samples and techniques taken into account, we could confirm that increased humidity causes significant changes in the cellulose chain and that lignin acts as an antioxidant as previously shown [31,32], and high lignin content in the paper could suppress some of the cellulose degradation pathways. This work was a continuation of our previous research on the ageing of papers produced in a neutral environment. Our previous publication [33] investigated the influence of lignin content on paper strength in a humidity environment, while this work focused on the chemical effects on ageing of paper.

## 2. Materials and Methods

### 2.1. Cellulosic Pulps

Pinewood (*Pinus sylvestris* L.) was utilised in this work. Pulps with a broad spectrum of cellulose content (74.5–94.1%) were prepared from the wood, as raw material, to compare the structural changes in lignocellulosic paper materials with the differential chemical composition. The chemical composition of pulps was established within the scope of earlier research [33].

Cellulosic pine pulps were prepared using the sulfate method, described by Modrzejewski et al. [34]. Active alkali (20–38% per batch) was added, and the water to the wood ratio (*v*/*w*) was 4. The dry weights (DWs) of all the materials were determined before pulping. The pulping processes were conducted in a 15 dm^3^ PD-114 stainless laboratory digester (Danex, Katowice, Poland) with temperature regulation (water jacket) and agitation (3 swings/min; swinging angle, 60°) mechanisms. The wood was suspended in an alkaline sulfate solution and heated. The maximum digestion temperature was 172 °C, and the heating time was 120 min. Cooking, at the maximum temperature, also lasted for 120 min. The temperature was thereafter decreased to 25 ± 5 °C with cold tap water. After delignification, the material was washed several times with demineralised water and incubated overnight in demineralised water to remove the residual alkali-soluble fractions. Next, the solids were disintegrated for 3 min in a laboratory JAC SHPD28D propeller pulp disintegrator (Danex, Katowice, Poland), after which the fibres were screened with a PS-114 membrane screener (Danex, Katowice, Poland) equipped with a 0.2 mm gap screen. After screening, the pulps were dried for 48 h at ambient temperature. The dry pulps were stored in hermetically sealed vials until they were utilised for further experiments.

The residual lignin content, expressed as the Kappa number (ISO 302:2015) of the pulps, was also determined. Cellulose was quantified as alpha-cellulose, according to the Tappi T203 standard (alpha-, beta-, and gamma-cellulose in pulp). All the chemical analyses were performed in triplicate for each pulp.

### 2.2. Nomenclature of the Paper Samples and Sheets

In this work, as well as presented in the tables and figures, the nomenclature of the samples was based on the Kappa number, determined for each pulp, which contained in the range 19–90.

The pulps were employed to prepare laboratory-scale test sheets. Before processing, the pulps were soaked in water for 24 h. Next, they were treated in the aforementioned pulp disintegrator, following PN EN ISO 5263-1 (2006), at 23,000 revolutions. Further, they were refined in a JAC PFID12X PFI mill (Danex, Katowice, Poland) with a single batch comprising 22.5 g of dry pulp according to PN-EN ISO 5264-2 (2011). The cellulosic pulps were refined to 30°SR, where they exhibited maximum strength and could be easily dehydrated. The Schopper–Riegler freeness was measured with a Schopper–Riegler apparatus (Danex, Katowice, Poland) according to PN-EN ISO 5267-1 (2002). Next, the sheets of paper were formed in a Rapid-Köthen class apparatus according to PN-EN ISO 5269-2 (2007). Each laboratory paper sheet was specified to have a base weight of 80 g/m^2^. Only the sheets with base weights in the range 79–81 g/m^2^ were considered for the ageing tests.

### 2.3. Ageing Tests

The samples of the obtained papers with different delignification degrees were artificially aged under two ageing conditions. One test was conducted in a dry atmosphere, at 90 °C, employing a BMT Venticell laboratory dryer (“DRY” method). Next, a parallel set of samples was also aged according to the D6819 American Society for Testing and Materials (ASTM) standard (2007), at an elevated temperature, in the presence of water vapour (90 °C and RH 59% (“WET” method)) in a climatic chamber (Memmert HCP246). During the ageing in both types of chambers, a large volume of the degradation products diffused. The ageing of the sample lasted 0–90 days. The samples were collected after 48 and 90 days of ageing, employing both WET and DRY methods.

### 2.4. Characterisation of Cellulose in the Paper Sheets

#### 2.4.1. Determination of the Molecular Weight Distributions by SEC

The analysis of the molecular weights of cellulose was conducted employing the derivative of the original samples, cellulose tricarbanilate (CTC), which is soluble in tetrahydrofuran (THF). The DP and molecular weight distributions of the papers were determined by SEC following the method described in previous works [35,36,37]. The chromatographic configuration consisted of the Waters chromatographic system, equipped with an isocratic pump 1515, an autosampler 717+, a column oven, dual λ absorbance detector 2487 (254 nm), a multiple angle laser-light scattering (MALLS) detector (Dawn Heleos, Wyatt Technology, Hollister Ave, Santa Barbara, CA, US, working at 658 nm), and a differential refractive index detector (Optilab T-rEX, Wyatt Technology, working at 658 nm), which functioned as a concentration-sensitive detector. The separation was performed, employing a set of two 25 cm × 1 cm mixed-bed polydivinylbenzene columns (Jordi), which was thermostated at 35 °C. THF (HPLC grade, J. T. Baker) was utilised as the mobile phase, at a flow rate of 1.0 cm^3^/min. The Astra 6.1.1.17 (Wyatt Technology, Hollister Ave, Santa Barbara, CA, US) software was employed to process and analyse the chromatographic and MALL’s data. Molecular weights and DP values were averaged from 4 measurements: 2 injections from 2 separate batches of paper.

#### 2.4.2. Diffuse Reflectance Infrared Fourier-Transform Spectroscopy (DRIFTS)

DRIFT spectra were obtained on a THERMO/Nicolet 5700 spectrometer equipped with an MCT/A detector with a Harrick Praying Mantis appliance. A piece of the sample (diameter ca. 5 mm) was placed in the Harrick Praying Mantis appliance chamber, which was continuously purged with dry helium (ca. 15 cm^3^/min). To remove water, the temperature of the Harrick chamber was set to 110 °C for 10 s. Before the measurements, the temperature was reduced back to 30 °C, at which the spectra were recorded. Since the analysed samples were characterised with different lignin contents, an attempt was made to obtain the DRIFT spectrum of pure lignin. Because pure lignin is almost black and highly absorbs incident IR light, the recording of its IR spectrum was extremely challenging. Moreover, lignin is hygroscopic and susceptible to thermal decomposition and, hence, it could not be heated before the measurement (it is conducted in the case of paper samples). Therefore, before the DRIFT analyses, pure lignin powder was diluted in potassium bromide (KBr), and the prepared powder solution was placed in the Harrick Praying Mantis chamber.

For the semi-quantitative comparisons of the samples with different Kappa value, the spectra were normalised by the internal standard method (integral of the band between 2.800 and 3.000 cm^−1^), described in our previous works [18,19,25], and presented as a standardised absorbance (Astd). The degradation process was investigated in the range of 1500–1900 cm^−1^ where the carbonyl groups evolved.

#### 2.4.3. UV–Vis Spectroscopy

The UV–Vis spectra were measured, employing the Avantes setup, which consisted of an Avalight-DH-S-Bal combined light source (deuterium and halogen lamps); AvaSphere-30-REFL integrating sphere (diameter, 30 mm, Spectralon covering, Apeldoorn, Netherlands); AvaSpec-2048 × 14-USB2 spectrometer with a CCD detector (2048 × 14 pixels); optical fibre with a diameter of 800 μm, channelling light from the source to the sphere; and optical fibre with a diameter of 800 μm, channelling light from the sphere to the spectrometer. The spectra were recorded in the range of 248–1050 nm with a resolution of 2.4 nm. Reflectance measurements were conducted twice for each sample. Further, the white (Rwb) and black (Rbb) spectra were also measured, and all the recorded spectra were normalised, adopting the white standard. Employing the original Kubelka–Munk theory, the spectra of each sample, measured in the UV-Vis range, were converted by equations into the reflectance spectra, R, of infinite thickness.

For semi-quantitative analysis, the oxidation indexes were calculated as follows: the band integral, at 200–800 nm, was calculated for each paper sample (unaged, aged for 48 and 90 days). For each sample characterised by a particular Kappa number, the resulting integral values were divided by the integrals of the unaged sample. The marker, denoted as OIUV, was considered as the general indicator of oxidation.

#### 2.4.4. X-ray Diffraction (XRD)

XRD experiments were performed on an X’Pert Pro MPD diffractometer (Philips, Almelo, Netherlands), equipped with a Johansson monochromator with a copper Kα1 line (λ = 1.5405 Å) and silicon position-sensitive X’Celerator detector. The measurements were conducted, employing the Bragg–Brentano θ–2θ geometry in the range of 2θ = 10°–40°, an increment of 0.008°, and time of 240 s for each angular step. During the measurements, a variable divergence slit was employed to obtain constant sensitivity within a whole range of 2θ. All diffraction spectra were recorded on an automatic divergence gap. This implied that the whole sample surface was illuminated throughout the measurement. The measured diffractograms were smoothened using the Savitzky–Golay method (~31 points) and the baseline was corrected using Essential FTIR software.

## 3. Results and Discussion

### 3.1. Impact of Accelerated Ageing on the Cellulose Depolymerisation Process: SEC Analysis

**Figure 1:**
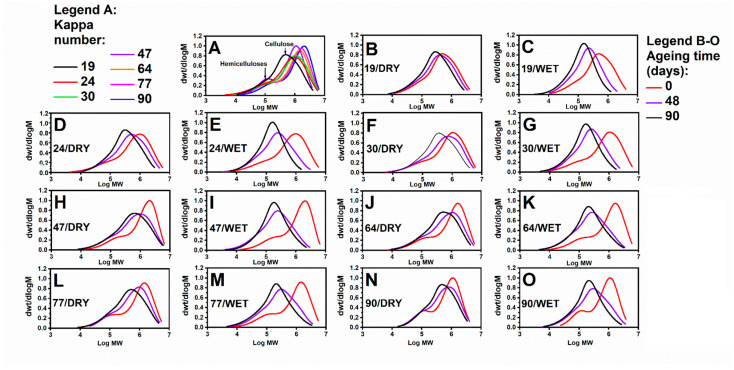
MMD curves of (**A**) unaged paper samples with different lignin contents and (**B**–**O**) papers containing different amounts of lignin aged in dry and humid atmospheres, DRY and WET, respectively.

The results obtained from the SEC are shown in Figure 1. By applying chromatographic separation, we could distinguish two main fractions through the molar mass distribution (MMD) curve: first, the low molar mass values that were attributed to the presence of hemicelluloses (armband) and, secondly, the high molar mass values that were attributed to the presence of cellulose-containing molecules (main band) (Figure 1A). The relative intensities of these two fractions are dependent on the relative hemicellulose and cellulose contents of a particular paper sample. Thus, for the sample with the lowest Kappa number, 19, the armband on the MMD curve is moderately noticeable (Figure 1B,C), while for the sample characterised with the highest Kappa number, 90, the armband exhibited a separated maximum at Log MW of about 5 (Figure 1N,O). Generally, a gradual decrease in the armband was observed in all the samples as ageing progressed. This might be due to the degradation of hemicelluloses. Moreover, as the ageing time was extended, the cellulose depolymerisation process also progressed significantly as expressed by a shift in the main band towards lower molar mass values and Log MW, consequently. Therefore, the cellulose and hemicellulose fractions could have overlapped for samples at the final stages of ageing.

**Figure 2:**
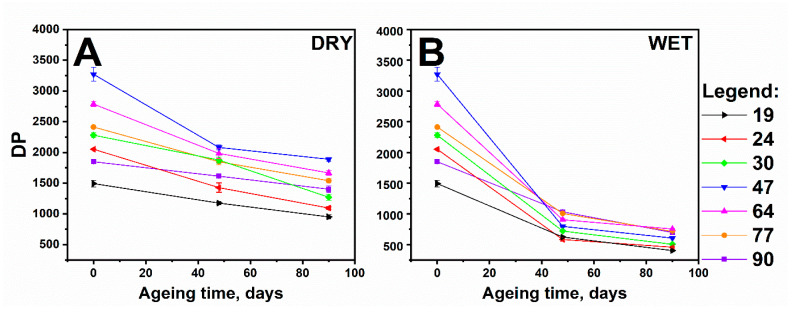
Degree of polymerisation (DP) values of cellulose and their changes with time, under different ageing conditions: (**A**) dry atmosphere and (**B**) humid atmosphere.

For the quantitative comparison of the trends, DPs were derived from the weight-averaged molar masses of cellulose, determined by SEC (Figure 2A,B). The DP values for the unaged samples are different among the samples because the raw wood material was subjected to different chemical treatments.

Depolymerisation largely occurred in the case of the samples, which were aged independently in the presence of water vapour in the lignin (Figure 2B). Accordingly, for the samples aged in dry air, the DP value did not fall below 1000 in any case (Figure 2A). This highlights the role of water in the depolymerisation of cellulose, i.e., water is a medium for protons during acidic hydrolysis (H_3_O^+^).

Because the initial DPs differed significantly among the samples, all the DP values obtained for the aged samples (after 48 and 90 days of ageing in dry and humid atmospheres) were normalised by dividing them by their corresponding initial DP values. Thus, the changes were expressed as percentage decreases from their initial values (Table 1).

The changes in DP during ageing in a chamber, both with dry air and controlled RH, may indicate a slow cellulose depolymerisation process for the samples with high Kappa numbers considering the percentage loss in the initial DP after 90 days of ageing.

Further, an increased lignin content could protect cellulose from depolymerisation, especially in a humid atmosphere. Considering that water molecules could be sources of radicals, we hypothesised that the polyphenols present in lignin may have captured the radicals and, therefore, reduced the number of glycosidic bond breakages in cellulose.

### 3.2. Impact of Accelerated Ageing on Cellulose and the Lignin Oxidation Process: DRIFT

**Figure 3:**
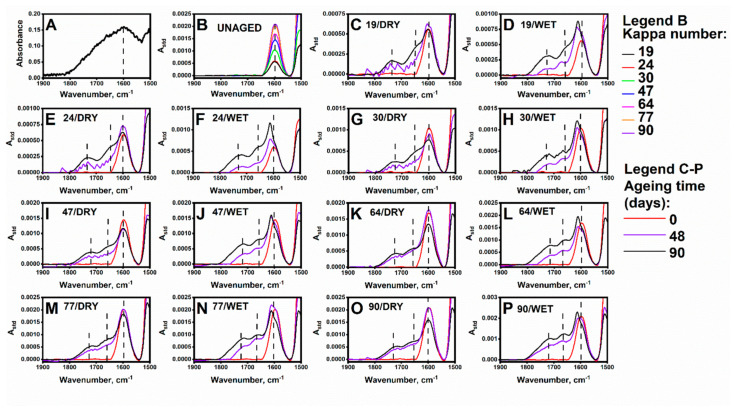
DRIFT spectra of (**A**) lignin, mixed with KBr; (**B**) unaged paper sheets with different Kappa numbers (spectra subjected to standardisation) (**C**–**P**).

The DRIFT results, obtained for the lignin and paper samples, are presented in Figure 3A–P. The interpretation of the lignin spectrum (Figure 3A) was complicated because of the high noise level. However, a broad scope in the spectral range of 1550–1800 cm^−1^ with a maximum at ~1600 cm^−1^ was well distinguished. This maximum could be assigned to the lignin aromatic ring vibrations and C=O stretching vibrations [38]. For the paper samples, as the Kappa number increased (and thus the lignin content), the intensity of the band around 1598 cm^−1^ evolved (Figure 3B). Therefore, the intensity of this band reflected the relative lignin content, as expected when comparing the different paper samples with various lignin concentrations.

Regarding the aged samples (Figure 3C–P), the gradual evolution of the new bonds were observed at ca. 1734 cm^−1^ and ca. 1661 cm^−1^. They are assignable to the lignin and hemicellulose components, e.g., the phenolic alcohols (coniferyl, sinapyl, *p*-coumaryl alcohols) and their degradation products (quinone derivatives). The intensities of those two bands (expressed as standardised absorbance) increased with increasing Kappa numbers. Notably, a difference in those band evolutions was observed, under the ageing conditions. The spectra of the paper samples aged under “DRY” conditions displayed bands with similar shapes after 48 and 90 days of ageing. Moreover, for the samples, aged in a “WET” atmosphere, significant broadening and growth in the intensity of the band at 1734 cm^−1^ were observed. Thus, it could be concluded that the rate of cellulose/lignin oxidation was slower in the absence of water. Generally, the evolution of the 1734 and ca. 1661 cm^−1^ bands was more pronounced when the samples were aged in the climatic chamber under “WET” conditions. Additionally, for the samples aged in the humid atmosphere, a shift in the band at 1600 cm^−1^ towards higher wavenumbers as the ageing time increased was more evident than in corresponding samples aged in the absence of water vapour. This shift is attributable to the –C=O stretching vibrations of aldehydes (coniferylaldehyde, sinapylaldehyde, and coumarylaldehyde) and ketones resulting from the oxidative degradation of lignin. It could be further deduced that the role of water in the oxidative degradation mechanism was at least two-fold: it acted as a transport medium for both protons in acidic hydrolysis (H_3_O^+^) and radicals in oxidation (OH^•^), and it acted as a plasticiser, which facilitated the access of cellulose and lignin to degradation agents.

### 3.3. Impact of Accelerated Ageing on the Cellulose Oxidation Process: UV–Vis Analysis

UV–Vis reflectance spectroscopy enabled the observation of the formation of the carbonyl groups. However, it was not employed on a larger scale. The limitation of the analysis is the low-characteristic spectra of the non-oxidised and oxidised papers, which lacked clearly formed bonds.

Three major bands were distinguished in the UV–Vis reflectance spectra of the unaged paper samples: 245, 280, and 335 nm. The band at ca. 240 nm was assigned to the hexenuronic acid groups [39]. The most intense band, at 280 nm, was assigned to lignin in the pulp [40], and the band at 335 nm could be assigned to the α-carbonyl structures and some conjugated structures. This band could also be attributed to the presence of coniferylaldehyde, which is a product of coniferyl alcohol oxidation and is a major contributor to the colour of lignin. Its absorption maximum is at 350 nm of the UV spectra of chemically treated and aged paper samples [41].

**Figure 4:**
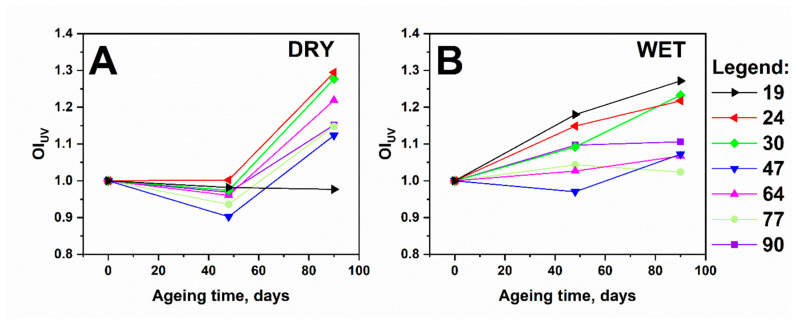
UV–Vis reflectance spectra of paper samples with different Kappa numbers, aged under different ageing conditions: (**A**) dry atmosphere and (**B**) humid atmosphere.

Observation of the OI_UV_ oxidation coefficient (Figure 4) indicated that the most changes occur in a series of samples aged in a humid atmosphere. Further, an evident growth in the OI_UV_ index was observed after 48 days and was still noticeable after 90 days. Regarding the samples aged in the dry atmosphere, a significant change in the oxidation index was noticed only after 90 days of ageing. This observation is consistent with the DRIFT results, which also indicated more evident formations of oxidation products during ageing in the humid atmosphere. No particular trend was observed when evaluating correlation of the lignin content with the extent of oxidation.

### 3.4. Impact of Accelerated Ageing on the Crystallinity of Cellulose

**Figure 5:**
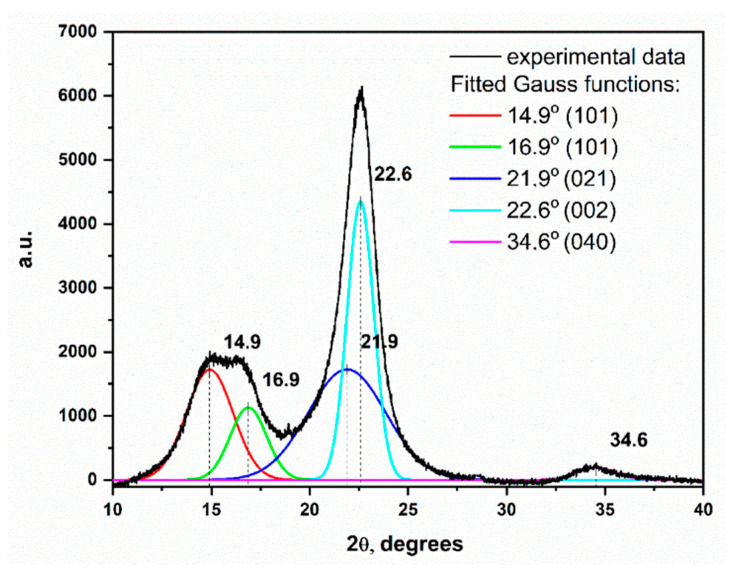
XRD pattern of model cellulose (Sigmacell) with fitted Gauss functions.

Diffraction patterns with fitted Gauss functions for the model cellulose sample are presented in Figure 5.

Typical cellulose, I, diffraction pattern also exhibited six characteristic reflections, which were assigned to the cellulose crystalline regions [42,43,44]. The diffractogram of the model sample of the microcrystalline cellulose powder exhibited all the characteristic reflections, namely (101), (10͞1), (021), (002), (040), of the cellulose structure, I, described in the literature [44]. In addition to the crystal reflections, the measured diffraction pattern was also affected by the amorphous phase of cellulose, which generated a broad peak with a maximum, at about 21° and a half-width of about 5°. The diffractograms of the samples with high cellulose content differed from those of microcrystalline cellulose with a more amorphous phase. The high concentration of lignin was manifested by obscuring the reflex (021) and raising the local minimum, at about 18°, relative to the background of the diffractogram. The diffractograms of lignin were observed as wide plateaus between 12 and 25°.

The determination of the crystallinity indexes required a comparison of the intensities of reflection with a value of 2θ, corresponding to the crystalline and amorphous phases. Two methods were employed to determine the crystallinity of the tested model paper samples [42]:

- CI_height_, calculated as the ratio of the peak height from the reflex (002) to the minimum height between the reflections (002) and (10͞1), is proportional to the amount of the amorphous phase, obtained by the formula: CI_height_ = (I002 − I_amorphous_)/I002 × 100%;
- CI_deconvolution_, calculated as the percentage of the peak areas were derived from the crystalline phase, (101), (10͞1), (002), (040), on the total surface of all the fitted Gaussian curves.

CI_height_ index was relatively easy to calculate, and this is probably the reason it is widely employed in the literature. However, it demonstrated several limitations:

- This factor did not accurately reflect the amount of the amorphous phase that was responsible for the formation of a wide peak with a maximum of about 21.9° while the intensity of the minimum, at about 18.5°, was employed to calculate CI_height_. An underestimation of the amount of amorphous phase is associated with the overlap of the reflex, derived from the amorphous phase with the most intense peak, derived from the crystalline phase (002) at about 22.6°. As a result, the sensitivity of the intensity of the measured I_amorphous_ to changes in the amount of the amorphous phase was low and further weakened by the contribution of the peak (10͞1) to the value of I_amorphous_. Accordingly, the CI_height_ index values, compared to other crystallinity indexes, were not very accurate.
- The number of crystalline phases was calculated based on the intensity of the peak (002): one of the four, observed on the diffractograms. In the case of the relative differences in the intensities of the reflections from the walls, (101), (10͞1), (002), (040), this could be a source of error.
- The reflections on the diffractograms of the lignocellulose samples were characterised by a large width, which changed with the progression of degradation. It also depended on the size of the crystallites and may vary under different measuring conditions. Therefore, the estimation of the amount of crystalline and amorphous phases in cellulose should depend on the calculation of the peak areas derived from the individual phase reflections and not on the comparison of the relative peak heights (expressed as intensities).

The calculation of the CI_deconvolution_ index required an adjustment to the experimentally obtained diffractograms of several Gauss functions. One Gauss function and an additional one, simulating the reflex originating from the amorphous phase, were fitted for each considered crystal reflex. The CI_deconvolution_ index was expressed as a percentage of the surface of the crystal reflections concerning the surface of all the reflections. In the literature, five Gauss functions are fitted to calculate CI_deconvolution_ (for phase reflections: (101), (10͞1), amorphous (021), (002), (040)) or six ((101), (10͞1), amorphous (040), (002), (040) and (021), at 2θ 21°) [42].

The deconvolution of the diffractograms, measured for the studied paper samples with variable lignin contents, was performed employing five Gauss functions (Figure 5). Of note, for the papers containing lignin, the calculated crystallinity indexes would be subject to an error related to the presence of lignin. Hence, it must be assumed that the underestimation of the degree of crystallinity might be greater with an increase in lignin content. Both the lignin and amorphous cellulose phases afforded a nonspecific signal, typical of the amorphous phase and, thus, the determined CI_deconvolution_ values would be most likely underestimated.

Since the XRD measurements were time-consuming, only one XRD measurement was performed per sample. Considering the uncertainty of the measurement, paper heterogeneity (estimated at a low level) and the lack of a distinguished direction of fibre arrangement relative to the incident X-ray beam, the crystallinity indexes determined by the deconvolution method afforded a relative standard deviation of <1%. Notably, the values of the crystallinity indexes were underestimated because of the presence of amorphous lignin. Although the CI_deconvolution_ index is more accurate than CI_height_, it did not allow the determination of the absolute contents of the crystalline and amorphous phases of cellulose. However, it was possible to employ it for tracking of the trends of changes in crystallinity.

**Figure 6:**
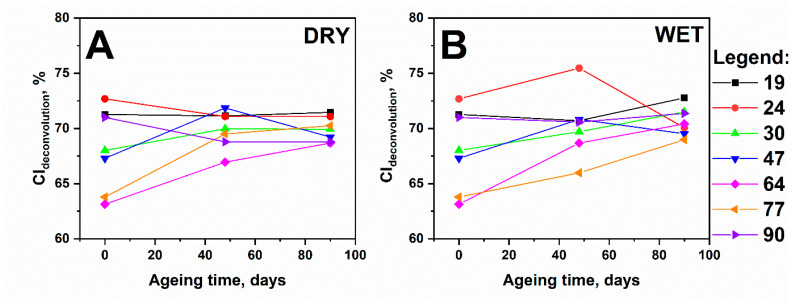
Values of crystallinity indexes and their changes in time under different ageing conditions: (**A**) dry atmosphere and (**B**) humid atmosphere.

The values of the crystallinity coefficients for the samples containing lignin (Figure 6) reflected the content of the cellulose crystalline phase in the paper sample but not in the total cellulose mass because the signal from the amorphous phase of cellulose and lignin was indistinguishable.

Recrystallisation is a physical degradation process that could be accelerated by both oxidation and hydrolysis. Figure 6 presents the values of the crystallinity coefficients as a function of ageing time, determined for the unaged and aged samples after 48 and 90 days.

For most of these samples, a slight increase in the CI_deconvolution_ value could be observed. The changes in the values of the crystallinity index with ageing time are irregular, which may indicate that the random error of the CI_deconvolution_ calculation method was comparable to the observed changes. Noticeable changes in crystallinity were observed for the series of samples aged in a humid atmosphere, while the changes were slightly lower for the samples aged in a dry atmosphere. A ventilated atmosphere with RH, oscillating around 59%, could facilitate the repeated penetration and desorption of moisture, thereby causing permanent changes in cellulose crystallinity. Consequently, when ageing in a dry atmosphere, this process was very slow because of the stabilisation of the atmosphere and the minimum water content in the environment. Additionally, water is a plasticiser, which changes the geometry of hydrogen bonds, thus causing the approximation and formation of new hydrogen bonds between macrofibrils.

**Figure 7:**
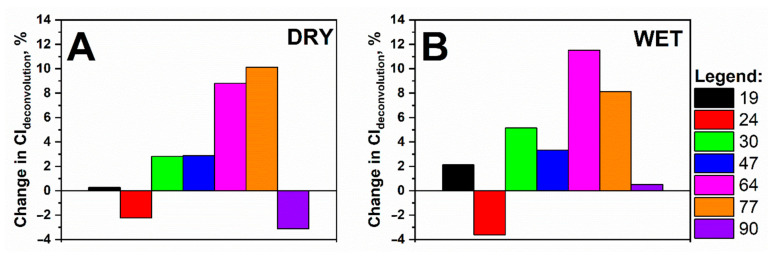
Changes in crystallinity indexes, observed for the cellulose/lignin samples, under different ageing conditions: (**A**) dry atmosphere and (**B**) humid atmosphere.

Comparing the relative changes in the crystallinity indexes (Figure 7), no specific dependence on the lignin content or ageing conditions was observed. Generally, it was observed that a greater change in the CI values was observed after the ageing of samples with a lignin content of between 9.6% to 11.5% (samples with Kappa numbers 64 and 77, respectively). The samples with lower lignin contents underwent moderate (samples with Kappa numbers 30 and 47) changes in their crystallinity. Regarding the samples with the lowest and highest lignin content (2.9%; Kappa number 19; 13.5%; Kappa number 90, respectively), crystallinity change could depend on the ageing conditions. In a dry atmosphere, the samples with low lignin contents exhibited only 0.27% change in crystallinity, while in humid air, an almost 10-fold increase in the relative change of CI was observed (Figure 7A,B).

Regarding the samples with the highest Kappa number, we observed a trend, which was a reverse of that observed in the samples with Kappa number 19. When the sample was subjected to dry air conditions, the crystallinity value dropped by ~3.13% after 90 days of ageing. However, in the case of ageing in the presence of water vapour, this change was significantly lower and was equal to ~0.51%.

Both hydrolysis and oxidation occurred mainly in the amorphous areas of cellulose considering the changes in the crystallinity index values of a series of samples aged in humid and dry atmospheres (Figure 7). For the sample with the lowest Kappa number, the crystallinity index increased in both ageing series, while for the sample with Kappa number 90, this increase was only observed for the series with ageing in a humid atmosphere. Thus, it could be assumed that the rate of hydrolysis decreased when the crystallinity index increased, and vice versa. When the percentage of the crystalline phase decreased, the course of the hydrolysis process became likelier. This corresponds to the trends obtained by SEC for samples aged in humid air (Figure 2, Table 1).

## 4. Conclusions

This work aimed at resolving two issues: First, it studied how humidity in the air affected the cellulose structure in papers containing lignin and cellulose. Secondly, it assessed the extent to which the instrumental methods were applicable and beneficial to study of the aforementioned challenges and for which lignin contents.

Employing chromatographic, spectroscopic, and diffraction techniques, it was possible to tackle the structural changes in cellulose and lignin caused by artificial ageing in dry and humid atmospheres. Significant changes in the cellulose chain lengths were observed, and these changes were associated with humidity during ageing. The formation of new functional groups was confirmed using two spectroscopic techniques, DRIFT and UV–Vis. For lignocellulosic materials, DRIFT could also deliver complex information about the degradation products of lignin. The SEC technique demonstrated the role of lignin as a protective shield for cellulose in the paper. Additionally, the XRD technique was employed to observe the influence of water vapour on the crystalline structure of cellulose and its possible implications on other degradation pathways. Overall, it was observed that degradation processes are faster in humid atmosphere possibly because of the protons and OH^•^ radicals supplied by water molecules. Interestingly, a high concentration of lignin in the paper could suppress some of the degradation pathways because of the polyphenols, which acted as radical scavengers.

Regarding the significance of the above techniques for testing lignocellulosic paper materials with different Kappa numbers, we demonstrated that paper with a broad spectrum of lignin content could be studied using non-invasive (UV–Vis, X-ray) or nondestructive (UV–Vis, X-ray, DRIFT) methods. Further, to the best of our knowledge, this is one of the few works that has utilised IR spectroscopy to study lignin and lignocellulosic materials. The SEC technique involves destruction of the sample. However, because of the small amount of sample required, SEC could be considered as a microdestructive technique. More significantly, the SEC technique was the most appropriate method to study DP of lignocellulose materials because it afforded information on the whole molar mass distributions during the processes leading to cellulose depolymerisation and was not limited to pure cellulose samples. Importantly, all the techniques afforded complementary information and availed a comprehensive picture of the structural changes in lignocellulosic paper materials for a broad range of Kappa numbers.

## Figures and Tables

**Table 1:** Decrease in DP values after 48 and 90 days of ageing.

	Percentage Fall of DP upon Ageing			
Kappa Number [–]	After 48 Days		After 90 Days	
	DRY	WET	DRY	WET
19	21.35	58.30	36.28	73.09
24	30.49	71.60	46.66	77.74
30	17.70	68.45	44.35	77.91
47	36.36	75.68	42.29	81.48
64	28.80	67.58	40.24	72.96
77	23.40	58.30	36.23	70.39
90	12.76	44.11	24.22	62.27

## Data Availability

The datasets generated during and/or analysed during the current study are available from the corresponding author on reasonable request.
